# The structure and regulation of Cullin 2 based E3 ubiquitin ligases and their biological functions

**DOI:** 10.1186/s13008-016-0020-7

**Published:** 2016-05-23

**Authors:** Weijia Cai, Haifeng Yang

**Affiliations:** Department of Pathology and Cell Biology, Thomas Jefferson University, Philadelphia, PA 19107 USA

**Keywords:** Cullin-2, Elongin, E3 ubiquitin ligase, VHL, Hypoxia inducible factor, Rack1, E4orf6, Viral host defense

## Abstract

**Background:**

Cullin-RING E3 ubiquitin ligase complexes play a central role in targeting cellular proteins for ubiquitination-dependent protein turnover through 26S proteasome. Cullin-2 is a member of the Cullin family, and it serves as a scaffold protein for Elongin B and C, Rbx1 and various substrate recognition receptors to form E3 ubiquitin ligases.

**Main body of the abstract:**

First, the composition, structure and the regulation of Cullin-2 based E3 ubiquitin ligases were introduced. Then the targets, the biological functions of complexes that use VHL, Lrr-1, Fem1b, Prame, Zyg-11, BAF250, Rack1 as substrate targeting subunits were described, and their involvement in diseases was discussed. A small molecule inhibitor of Cullins as a potential anti-cancer drug was introduced. Furthermore, proteins with VHL box that might bind to Cullin-2 were described. Finally, how different viral proteins form E3 ubiquitin ligase complexes with Cullin-2 to counter host viral defense were explained.

**Conclusions:**

Cullin-2 based E3 ubiquitin ligases, using many different substrate recognition receptors, recognize a number of substrates and regulate their protein stability. These complexes play critical roles in biological processes and diseases such as cancer, germline differentiation and viral defense. Through the better understanding of their biology, we can devise and develop new therapeutic strategies to treat cancers, inherited diseases and viral infections.

## Background

Cullin-RING E3 ubiquitin ligase complexes (CRLs) play a central role in targeting cellular proteins for ubiquitination-dependent protein turnover through 26S proteasome [1]. Cullin-2 (Cul2), a member of Cullin family proteins, is encoded by *CUL2*. Cul2 functions as a scaffold protein to form CRLs that belong to the Elongin B and C-Cul2 or Cul5-SOCS box protein (ECS) family [2]. In CRL2 complexes, Cul2 assembles with RING protein (Rbx1) (also known as Roc1) as RING finger protein, Elongin B and C proteins as adapter proteins and various substrate recognition receptors [2, 3].

Cul2 is different from other most Cullins, which are evolutionarily conserved from yeast to human. Cul2 is present only in multi-cellular organisms and plays a particular function [4]. The most well-known CRL2 substrate recognition receptor is the tumor suppressor protein VHL that is mutated in von Hippel–Lindau (VHL) syndrome, a rare hereditary cancer syndrome [5]. Germline *VHL* mutations usually disrupt the interaction between VHL and Elongin B and C, and inactivate the VHL-Elongin B/C-Cullin-2 E3 ligase [6]. CRL2^VHL^ complex-dependent degradation of the α subunits of hypoxia inducible factor (HIFα) is the most studied role of CRL2 ubiquitin ligase in tumorigenesis [7, 8]. In addition, CRL2 ligases are involved in other cellular processes including germline development and viral infection. This review will go over the structure and regulations of CRL2 ligases, their substrate recognition receptors and their numerous substrates, and discuss their involvement in biological processes and diseases.

## Main text

### Structure and regulation

**Fig. 1:**
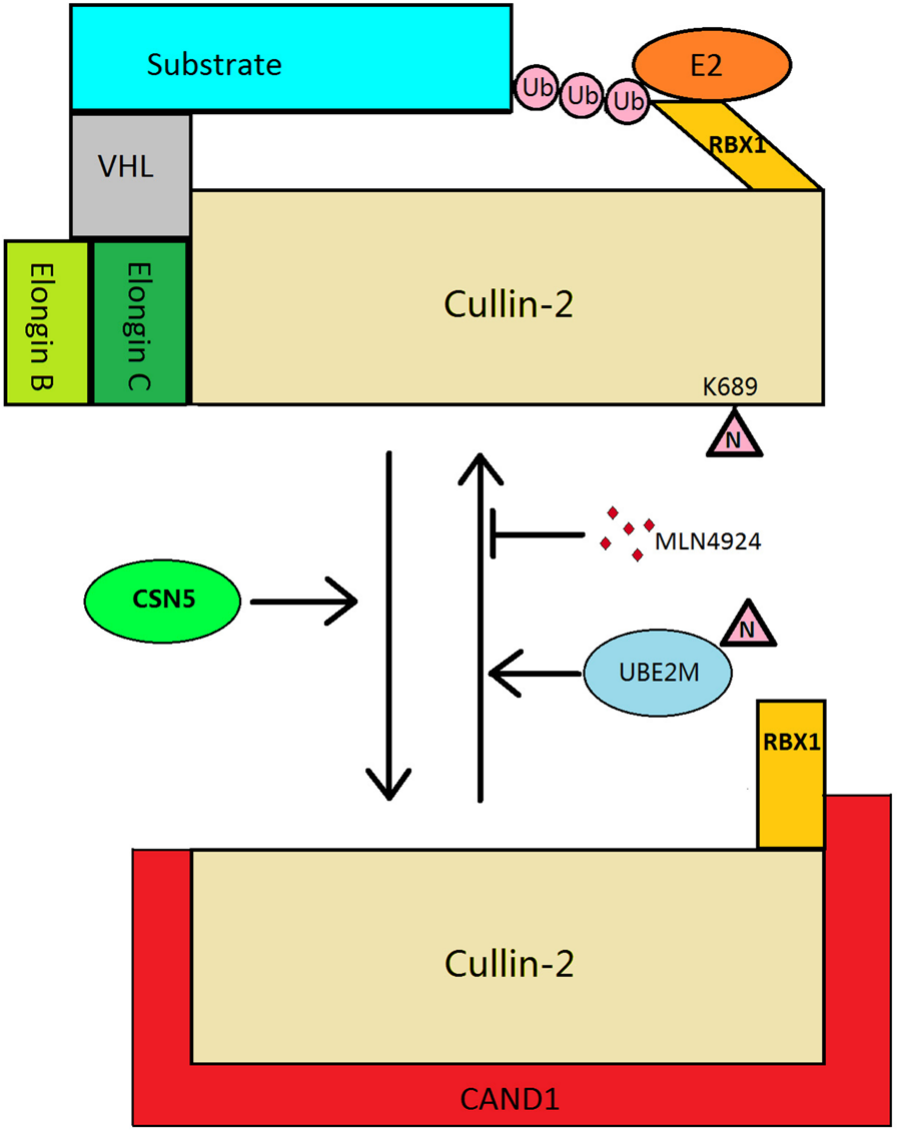
Structure of CRL2^VHL^ complex and Nedd8-mediated regulation of CRL2 activity. Cul2 is the scaffold protein that binds to Rbx1, Elongin C and VHL directly. Neddylation on lysine 689 of cullin-2 dissociates Cand1, which allows the Cul2 to bind to Elongin B, C and VHL, facilitates appropriate conformation of Rbx1 and promotes ubiquitination on substrate proteins. Ube2m promotes neddylation of Cul2 and increases CRL2 activity, whereas CSN5 and inhibitor inhibit CRL2 activity. *Ub* ubiquitin, *N* Nedd8

Similar to other Cullins, Cul2 contains an evolutionary conserved Cullin homology (CH) domain at its C-terminus. The CH domain was found to interact with Rbx1, which further recruits E2 ubiquitin conjugating enzymes [9] (Fig. 1). The N-terminus of Cul2 was responsible for interacting with Elongin B and C and various substrate recognition receptors (Fig. 1). These receptors usually contained a special domain called VHL-box [10].

Elongin B and C proteins were originally found as two regulatory subunits of the Elongin complex, which was a positive regulator of RNA polymerase II and increased the rate of mRNA elongation by suppressing transient pausing along the DNA template. Elongin B and C bound to each other and enhanced the transcriptional activity of the other component of Elongin complex, Elongin A [4–6]. Elongin B and C were later found to bind to Cul2 or Cullin-5 (Cul5) and serve as adapter components of ECS ubiquitin ligases [11–13].

**Fig. 2:**
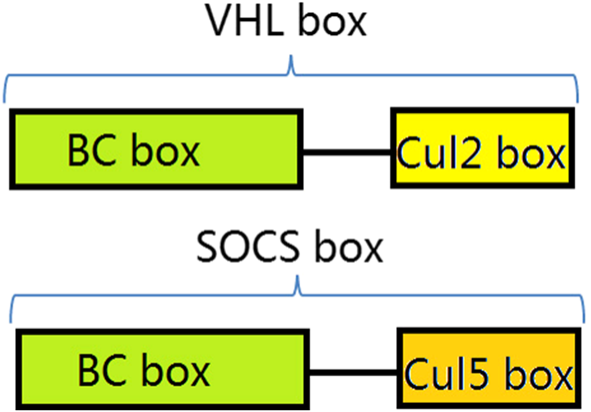
The compositions of VHL box and SOCS box. VHL box is made up of a BC box and a Cullin2 box. SOCS box consists of a BC box and a Cullin5 box

VHL and other Cul2-Rbx1 interacting proteins such as Leucine-Rich Repeat protein-1 (LRR-1) and Feminization-1 (FEM-1) have a region of homology called the VHL box (Fig. 2). This box contained both a BC box [14] (consensus sequence: (S,T,P)LXXX(C,S,A)XXXϕ, with ϕ meaning a hydrophobic amino acid), which bound to Elongin B and C, and a Cullin 2 box (consensus sequence: ϕP*XX*ϕ*XXX*ϕ), which was responsible for binding to Cul2. Detailed alignment that defined VHL box could be found in Mahrour et al. [10]. VHL box was very similar to Suppressor Of Cytokine Signaling (SOCS) box, which also contained a BC box and a Cullin 5 box (Fig. 2). Although both VHL box proteins and SOCS box proteins used Elongin B and C as an adaptor, they bound to different Cullins. The different Cullin boxes determined the binding specificity to Cul2 and Cul5 [14–16].

A recent paper described the crystal structure of a CRL2 complex composed of VHL, Elongin B and C and the N-terminus of Cul2 [17]. It showed that in many ways CRL2 structure were different from these of CRL1 or CRL5 complex. The CRL2 complex assumed a tripod shape, with Elongin C located in the center and the other components at the ends. Cul2 bound to the interface between VHL and Elongin C through hydrophobic and electrostatic interactions. Cul2 binding induced a structuring of Elongin C loop (residue 48–57) which made contact with Cul2. The same loop was not structured in the VHL-Elongin BC complex [18]. Different from Cul5, the N-terminal extension of Cul2 played a critical role in binding to Elongin C. For example, residue L3 of Cul2 inserted into a hydrophobic pocket of Elongin C. L3G mutant of Cul2 drastically reduced the interaction between Cul2 and VHL-Elongin BC complex. Consistently, the N-terminal extension was highly conserved across all Cullin-2 orthologs. Importantly, not only the Cullin 2 box was critical for Cul2-VHL interaction [16], the BC box on VHL also made critical contact with Cul2 via hydrogen bonds and salt bridge interactions [17].

Similar to other Cullin family members, Cul2 contained a neddylation site close to the RING protein (Rbx1) binding site [19]. Auto-neddylation of Cullin by Rbx1 induced conformational change at C-terminus, resulting in stabilization of an optimal Rbx1 position and activation of CRL ubiquitin transfer activity [20–22]. NEDD8-conjugating enzyme Ube2m (also known as Ubc12) promoted neddylation of Cullin 1-4 through Rbx1, whereas Ube2f neddylated Cullin 5 through Rbx2 [23]. Conversely, deneddylation by COP9 signalosome complex subunit 5 (Csn5) or a small molecule inhibitor of NEDD8-activating enzyme (MLN4924) [24] led to binding of Cullin-Associated and Neddylation-Dissociated 1 (Cand1) to Cullins. This binding sterically inhibited the interaction between Cullin and adaptor proteins, and impaired Rbx1-mediated E2 ubiquitin activation [24–30] (Fig. 1). Interestingly, engagement of substrates to CRL complex could induce Cullin neddylation [31, 32]. This ‘substrate-mediated neddylation’ was recently reported to be mediated by Defective in Cullin Neddylation 1 (Dcnl1) [33]. Dcnl1 was the human homolog of Dcn1 in *Saccharomyces cerevisiae*, also known as Sccro or Dcun1d1, which was an E3 Nedd8 ligase that promoted Cullin neddylation with Rbx1 [34–37]. Interaction between VHL and its substrate HIF1α promoted the recruitment of Dcnl1 to trigger Cul2 neddylation, and consequently HIF1α ubiquitination and proteasomal degradation [33].

### Different CRL2 E3 ubiquitin ligase complexes

There are a number of CRL2 complexes that are confirmed as functional E3 ubiquitin ligases. They can be divided into two groups: cellular CRL2 complexes that are derived from cellular proteins, and viral CRL2 complexes that contain viral proteins. Known CRL2 E3 complex and their substrates are summarized in Table 1.

**Table 1:** Known CRL2 complexes and their substrates

Ubiquitin ligase	Interaction domain	Substrates	Reference
VHL	VHL box	HIFα	[1–5]
		Spry2	[6]
		Rpb1	[7, 8]
		Rpb7	[9]
		Atypical PKC (PKCλ and ζII)	[10, 11]
		EGFR	[12]
LRR-1	VHL box	CKI-1 (C. elegans)	[13–15]
		p21CIP	[15]
		HTP3 (C. elegans)	[16]
FEM1/Fem1B	VHL box	TRA-1(C. elegans)	[17]
		Gli1	[18]
		Ankrd37 (mouse)	[19]
Prame	VHL box	unknown	[20, 21]
ZYG-11/ZER1	VHL box	unknown	[22, 23]
BAF250/Osa/BAF250b	BC box	H2B	[24]
Rack1	BC box-like	HIF-1α	[25]
		BimEL	[26]
Ad E4orf6	BC box	p53	[27–30]
		Mre11	[29–31]
		DNA ligase IV	[29, 30, 32]
		Intergrin α3	[29, 30, 33]
		TOPBP1	[29, 34]
EBV Bzlf1	Cul2 box	p53	[35–37]
HPV16 E7	incomplete Cul2 box	pRB	[38–41]
BIV Vif	BC box	A3Z2-Z3 (Bos taurus)	[42, 43]
Unknown	Unknown	RhoB	[44]

#### CRL2^VHL^ complex

Von Hippel–Lindau (VHL) syndrome was first described separately by von Hippel in 1911 and by Lindau in 1926 [5]. It was characterized by the development of multiple vascular tumors and was caused by a mutation of both alleles of the VHL gene located on the short arm of chromosome 3 [38]. VHL was a 213 amino acid protein product of the *VHL* tumor suppressor gene. Most germline *VHL* mutations were missense alterations that produced mutated VHL proteins that lost ability to bind to Elongin B and C [39, 40]. Further study showed that VHL formed a complex with Cul2, Elongin B and C and Rbx1, and had E3 ubiquitin ligase activity [6, 9, 41]. The CRL2 ligase complex could bind to HIFα through the β domain of VHL, promote ubiquitination and proteasomal degradation of HIFα [42, 43]. HIFα family consisted of three members, HIF1α, HIF2α and HIF3α. They were unstable subunit of HIF complex, and formed the HIF transcriptional factor with constitutively expressed HIF1β, also called Aryl Hydrocarbon Receptor Nuclear Translocator (ARNT), to regulate gene expressions [7]. HIF downstream target genes [44] include vascular endothelial growth factor A (VEGFA) [45, 46], solute carrier family 2 member 1 (SLC2A1, which was also called GLUT1), and platelet-derived growth factor-β (PDGFB) [47], which were known to drive cell growth and proliferation of microvascular vessels in VHL syndrome [42].

The HIF transcriptional activity is tightly regulated by oxygen concentration. Under normal oxygen tension (normoxia), two key proline residues in the oxygen-dependent degradation domain of HIFα were hydroxylated by HIF prolyl hydroxylases (PHD1–3). Hydroxylated HIFα provided a binding signal for the β-domain of VHL [43, 48–53]. Consequently HIFα was poly-ubiquitinated by CRL2^VHL^ E3 ubiquitin ligase and degraded by the proteasome. When oxygen was taken away (hypoxia), HIFα was produced but not hydroxylated by PHDs, so it escaped recognition by VHL. As a consequence it would accumulate, form HIF, and activate the transcriptional program to respond to hypoxia [8]. Any other conditions that disrupt the functions of PHDs or VHL will also lead to HIF stabilization and the activation of HIF pathway. In hereditary VHL disease, mutations and loss of heterozygosity (LOH) at the *VHL* locus in the cancer cells inactivates VHL and results in a constitutively high level of HIFα even in the presence of oxygen. The activated HIF targets can have opposing effects on tumor growth [54, 55], but the overall activity of the constitutively active HIF pathway is the major oncogenic force that drives tumorigenesis and tumor growth. It was known to cause many manifestation of VHL disease such as clear cell renal cell carcinoma, hemangioblastoma and pheochromocytoma [7], and the partial blockage of HIF pathway by anti-angiogenesis drugs produced significant clinical benefits [56]. Currently, five drugs targeting VEGFA (bevacizumab) or its receptors (sunitinib, sorafenib, pazopanib and axitinib) were approved by FDA for the treatment of renal cell carcinoma (RCC). The median survival of advanced RCC patients had increased from less than 1 year (receiving cytokine IFN-alpha) to nearly 2 year (receiving targeted therapies) during the last decade [57].

Although HIFα is the most studied VHL substrate and probably the most important one, CRL2^VHL^ also recognizes and mediates the degradation of many other substrates. Similar to HIFα, Sprouty2 (Spry2), a protein that regulates cell migration and proliferation in response to a number of growth factors, was also hydroxylated by PHD at normoxia and recognized by VHL for degradation. Increased cellular level of Spry2 after silencing PHDs or VHL inhibited human fibroblast growth factor-elicited activation of ERK1/2 [58]. Rpb1 is the largest subunit of RNA polymerase II. It is also the enzymatic subunit of the complex, synthesizing cellular mRNAs [59]. Rpb1 contains an LGQLAP motif that bears sequence and structural similarity to a VHL-binding sequence in HIF1α [60]. Similar to HIFα, the proline P1465 within the motif of Rpb1 was hydroxylated under oxidative stress [60]. Proline 1465 hydroxylation by PHD1 and the further recognition by VHL was required for oxidative stress-induced Ser5 phosphorylation of Rpb1, the poly-ubiquitination of Rpb1 and the recruitment of Rpb1 to the DNA, which stimulated formation of tumors by VHL^+^ cells [59]. In a different cell line, Rpb1 was poly-ubiquitinated by VHL and degraded by proteasome [60]. Since the direct function of CRL2^VHL^ was ubiquitination, the next fate of Rpb1 was probably determined by different cellular context. hsRPB7, another subunit of RNA polymerase II, was also reported to be poly-ubiquitinated by VHL and degraded. Consequently, VHL suppressed hsRPB7-dependent VEGF expression [61]. Atypical protein kinase C (PKC) is made up of two members, PKCλ/ι (PKCι is the human homologue of mouse PKCλ) and PKCζ. In other reports, both PKCλ and PKCζII (a rapidly degraded variant of PKCζ) were poly-ubiquitinated by VHL and degraded [62, 63]. Epidermal growth factor receptor (EGFR) was also reported to be a target of CRL2^VHL^. VHL limited EGFR signaling by promoting c-Cbl-independent poly-ubiquitination and lysosome-independent degradation of the activated EGFR [64]. In addition, some E3-ligase independent functions of VHL were reported [65–68]. In these cases, VHL interacted with other proteins, regulated their functions, but did not promote their poly-ubiquitination and degradation. For example, VHL bound to NF-kappa B agonist Card9, promoted its phosphorylation by CK2 and inhibited NF-kappa B activity [64]. In particular, Lai et al. performed a series of proteomic analyses that identified many VHL-interacting proteins [66]. It’s a valuable resource for further investigation.

#### CRL2^LRR-1^ complex

Leucine-Rich Repeat protein-1 (LRR-1) in worm was found to have a VHL box and functioned as a substrate recognition receptor in a CRL2 complex [16, 69]. In *C. elegans,* the CRL2^LRR-1^ complex degraded the Cip/Kip CDK-inhibitor CKI-1 in nucleus to ensure a proper G1-phase cell cycle progression in the germ cells [69–71]. In human cells, the orthologous human CRL2^LRR1^ complex degraded the CDK-inhibitor p21^Cip1^, but did not regulate cell cycle because it only did so in the cytoplasm. Consequently, knockdown of Lrr1 resulted in increased cytoplasmic p21. This led to de-phosphorylation of cofilin through the inhibition of Rho/ROCK/LIMK pathway. The de-phosphorylated cofilin activated actin cytoskeleton remodeling and promoted cell motility [69].

CUL2 was highly expressed in the germline and in early embryos in *C. elegans* [70]. In *Drosophila melanogaster*, loss of function of CUL2 resulted in defects at the larval neuromuscular junction and aberrations in the development of female germ line [72]. Cul2 was also required to limit the number of motile cells in egg chambers [73] and for germline enclosure in testes [74]. Among several CRL2 complexes that were associated with germline development, CRL2^LRR-1^ complex was a critical one. LRR-1 null nematodes were defective in germ cell proliferation which resulted in animal sterility. Similar phenotype was observed in CUL2 null animals [71]. Since LRR-1 null germ cells arrested at G2/M stage, it was tested whether suppression of the DNA replication checkpoint would rescue the phenotype. It was discovered that the suppression of CHK-1 (Chk1 in humans for checkpoint kinase 1) or ATL-1 (ATR, Ataxia telangiectasia and Rad3 related) kinases, two core components of the DNA replication checkpoint pathway [75, 76], restored the fertility [71]. However, how LRR-1 or CUL2 deficiency caused hyper-activation of the DNA replication checkpoint pathway was still unknown. As CKI-1 suppression did not rescue the fertility phenotype of LRR-1 null animal, it was not the critical target for this phenotype [71].

In later steps of germ cell development, CRL2^LRR-1^ regulated the balance between mitotic proliferation and meiotic entry. It was probably because CRL2^LRR-1^ could regulate the degradation of unidentified meiotic promoting factors in the germline [77]. In nematode, CRL2^LRR-1^ inhibited the first steps of meiotic prophase through regulating the stability of HORMA-domain protein HTP-3, a key protein for loading synaptonemal complex components onto meiotic chromosomes [77]. Hence, CUL2 played multiple roles in the development of the germline in nematodes. Since Cul2 is conserved in multi-cellular organisms [4], the regulation mechanisms of germline development may be similar in other organisms as well [77, 78].

#### CRL2^FEM1B^ complex

Feminization-1 (FEM-1) was discovered to regulate apoptosis in the nematode sex determination pathway [79]. FEM-1 and its three homologs, Fem1a, Fem1b and Fem1c were found to contain a VHL-box, so theoretically they could interact with Cul2 [80]. Whereas FEM-1 and Fem1b were shown to target proteins for degradation, Fem1a and Fem1c were not confirmed as a component of CRL2 complex. In nematode, FEM-1 was found to target TRA-1 for ubiquitination [81]. TRA-1 was homologous to the mammalian Gli1 protein, an important transcription factor in Hedgehog signaling. Consistent with the worm data, Fem1b promoted ubiquitination and suppressed transcriptional activity of Gli1 in human [82]. Since Gli1 was an oncoprotein, Fem1b could be a tumor suppressor. Single nucleotide polymorphism (SNP) analysis revealed that Fem1b was associated with polycystic ovary syndrome [83]. In Fem1b-null mice insulin resistance was observed [84]. It was also reported to mediate apoptosis in human colon cancer cells [85] and served as a biomarker in mouse colon cancer model [86]. Fem1a was also implicated in polycystic ovary syndrome [83] and sonic hedgehog pathway hyperactivation in cancer stem cells in gastric cancer [87].

Mouse Fem1b induced ubiquitin-mediated degradation of Ankrd37, a protein that was enriched in mouse testis [88]. In addition, mouse Fem1b interacted with the homeodomain protein Nkx3.1, which was a pivotal regulator of prostate development. Both Fem1b and Nkx3.1 null mice show similar defects in prostate ductal morphogenesis [89]. These data indicate that Fem1b plays a conserved role in the generation of sexual dimorphism.

#### CRL2^PRAME^ complex

The human tumor antigen Preferentially Expressed Antigen in Melanoma (Prame) was frequently overexpressed in various cancers, and the high level expression was usually correlated with advanced stages and poor clinical outcomes in a wide variety of cancers [90]. The consensus LXXLL-binding domain at Prame‘s C terminus mediated interaction with the retinoic acid receptor (RAR), and Prame acted as a dominant repressor of RAR signaling and inhibited retinoic acid induced differentiation, growth arrest, and apoptosis [91]. At the N-terminus of Prame there was a VHL box, and it mediated the interaction with Elongin C and Cul2. Genome-wide chromatin immunoprecipitation experiments revealed that Prame associated with the transcription factor NFY at enhancers and transcriptionally active promoters. In addition, CRL2 complex were present together with Prame on chromatins [92]. Further analysis revealed that Prame interacted with OSGEP and LAGE3, two yeast proteins that were human orthologues of the ancient EKC/KEOPS complex. EKC/KEOPS complex was shown to play a role in telomeres maintenance, transcriptional regulation, and t^6^A modification of tRNAs [93, 94]. Furthermore, Prame recruited a CRL2 ubiquitin ligase to EKC complex on transcriptionally active chromatin [95]. The substrates of the E3 ligase activity of CRL2^PRAME^ complex are still unknown.

#### CRL2^ZYG-11^ complex

*ZYG*-*11* was identified as a gene that contributed to nematode zygote development in *C. elegans* [96]. Further analysis revealed a VHL box at the N-terminus of ZYG-11, and it was shown to bind to Elongin C and form complex with Cul2 [97]. Although the substrate(s) of CRL2^ZYG-11^complex was not identified, genetic analysis revealed that the complex was required for many functions of CUL2 in worm, such as the degradation of maternal cyclin B [97]. ZYG-11 homologues are restricted to metazoan. *C. elegans* has two ZYG11 family members, ZYG-11 and ZER-1. Both contain a VHL box and bind to Elongin C and CUL2. In human there are three ZYG11 family members, Zyg11a, Zyg11b and Zyg11bl. Only Zyg11b and Zyg11bl contained a VHL box and bound to Elongin C and Cul2 [97]. In human, Zyg11bl was found to be highly expressed in skeletal muscle and the testes [98], and it was specifically expressed in the cytoplasm of late pachytene spermatocytes and the round spermatids at meiotic division [98]. Although the substrate(s) was unknown, ZYG11 family members were proposed to function as substrate recognition receptors for CRL2 E3 complexes in the metazoan lineage [97].

#### CRL2^BAF250^ complex

Two isoforms of BAF250, BAF250a/ARID1A and BAF250b/ARID1B are defining components of human BAF complex. BAF complex and PBAF complex belong to SWI/SNF chromatin-remodeling complex, which remodels chromatin and facilitates DNA access by transcription factors and the transcription machinery [99]. Both BAF250a and BAF250b contained a BC box. BAF250b was shown to associate with Elongin B and C, Cul2 and Rbx1 to form an E3 ligase, which mono-ubiquitinated histone H2B on lysine 120 site [100]. Konckdown of BAF250a or BAF250b decreased levels of global H2B ubiqitination in human cell line. In addition, the BAF250 *Drosophila* homolog Osa mutant had reduced levels of mono-ubiquitinated H2B, and functioned synergistically with Cul2 in vivo [100]. These data suggest that BAF250 has an evolutionarily conserved function to regulate H2B ubiqutination as a component of CRL2 E3 ligase to promote transcription.

#### CRL2^RACK1^ complex

The Receptor for Activated C Kinase 1 (Rack1), a member of the tryptophan-aspartate repeat (WD-repeat) family proteins, was found to bind the N-terminus of Fem1b and poly-ubiquitinated Fem1b for proteasomal degradation in colon cancer cells [101]. Rack1 was also reported to act as an E3 ligase component to degrade ΔNp63α, a member of the p53 family [102]. Through the WD40 repeats, which contained an amino acid sequence similar to the VHL BC box, Rack1 was reported to bind to Elongin B and C and promoted the degradation of HIF1α in a HSP90-dependent but oxygen-independent manner [103]. In the presence of apoptotic agents, Rack1 mediated the degradation of Bcl-2-interacting mediator of cell death extra long (BimEL) through a CRL2 E3 ligase complex, and inhibited apoptosis in breast cancer cells [104]. The evidence suggests that Rack1 can be a component of CRL2 E3 complex and degrade target proteins via ubiquitin–proteasome pathway.

#### A CRL2 complex targeting RhoB

Since neddylation on Cullins was required for the activity of CRL complexes [20–22], a small molecule inhibitor of NEDD8-activating enzyme, MLN4924, could induce the accumulation of CRL substrates that lead to DNA damage, cell cycle defects, senescence, apoptosis and autophagy [24, 105–107]. It was tested by several phase I clinical trials because of its significant anticancer activity and relatively low toxicity in preclinical analyses [108–111]. A quantitative proteomic analysis identified RhoB as a target of CUL2-RBX1 complex [112]. The substrate-recognition subunit was not identified in this study. RhoB is a small GTPase and a member of Rho family. It acts as a tumor suppressor and is frequently down-regulated in various cancers. The MLN4924-induced accumulation of RhoB seemed to contribute significantly to the anticancer activity of this drug in liver cancer. A caveat is that MLN4924 impacts on many targets, so it is difficult to pinpoint the contribution of CRL2 to cancer development and treatment. Nonetheless, this highlights the potential therapeutic utility of targeting neddylation-CRL2-RhoB in liver cancer and other cancers.

### VHL box proteins

Several proteins have VHL box but were not confirmed as components of CRL2 complex. Their functions are summarized here, and their roles in CRL2 complex await further investigation.

Appbp2, the human homolog of *Drosophila* PAT1, also known as Ara67, was found to suppress androgen receptor (AR) transactivation through interrupting AR cytoplasmic-nuclear shuttling [113]. Appbp2 was found to be overexpressed through 17q23 amplification in neuroblastoma [114], ovarian clear cell adenocarcinomas [115] and desmoplastic medulloblastomas [116].

Kelch domain containing 2 (Klhdc2), also known as Hclp1, could serve a transcriptional co-repressor through its inhibitory interaction with the Lzip transcription factor [117]. Klhdc3, also known as Peas, is evolutionarily conserved from nematodes to mammals. Mouse Peas was found to be expressed in testis, particularly in the cytoplasm and meiotic chromatin of pachytene spermatocytes. It was suggested that Klhdc3 might be involved in the meiotic recombination process [118].

Zinc finger, SWIM-type containing 2 (Zswim2) was also known as MEKK1-related protein X (Mex), a testis-expressed protein. It contained an N-terminal SWIM (SWI2/SNF2 and MuDR) domain and two RING fingers separated by a ZZ zinc finger domain. Zswim2 was self-ubiquitinated as an E3 ubiquitin ligase and targeted for degradation through the proteasome pathway [119]. The SWIM domain was found to be critical for Zswim2 ubiquitination and was suggested to regulate death receptor-induced apoptosis in the testes. Zswim5 (also known as KIAA1511), Zswim6 and Zswim8 (also known as KIAA0913) all contained a VHL box and might play a similar role in E3 ligase complex, but this was not confirmed. Zswim5 displayed intense staining in gliomas but weak to modest staining in most other neoplasms [120]. Fyn-tyrosine-kinase-deficient mice had increased fearfulness and enhanced excitability. In the amygdala of Fyn-deficient mice, only Zswim6 expression was significantly lowered after administration of N-methyl-D-aspartate (NMDA) when compared with that in Fyn-proficient mice, suggesting that it might be a key mediator of the phenotype [121]. Zswim6 mutations were associated with acromelic frontonasal dysostosis, a rare disorder characterized by the craniofacial, brain and limb malformations. Zswim6 mutations might lead to the phenotypes through the disruption of Hedgehog signaling [122].

### Viral CRL2 E3 ligase complex

Viral infection activates host cell defense mechanisms, which will limit viral spread, inhibit viral replication and eliminate virus. Virus has developed various strategies to counter host cell defense and usurp the cellular machinery. One strategy is that viral protein formed E3 ubiquitin ligase complex to destroy host proteins. Several viral proteins that form CRL2 ligase complex had been reported to be indispensable for infection by adenovirus (Ad), Epstein–Barr virus (EBV), human papillomavirus (HPV) and bovine immunodeficiency virus (BIV).

Adenoviruses are linear double-stranded DNA viruses. They infect human and rodent cells, occasionally transform them and cause tumors in animal models [123]. The human adenovirus type 5 (Ad5) early region 4 from open reading frame 6 (E4orf6) contained three BC boxes and formed an E3 ubiqutin ligase complex with Cullin 5 (Cul5) [124, 125], whereas the human adenovirus type 12 (Ad12), type 16 (Ad16), type 40 (Ad40) and type41 (Ad41) formed complex with Cul2 [126, 127]. Adenoviral protein E1B55K associated with the E4orf6 protein and recognized substrate to be degraded by ubiquitin–proteasome pathway [124, 125]. In this complex, E4orf6 was believed to recruit Cul2 or Cul5 as an adaptor protein, whereas E1B55K was believed to act as a substrate recognition receptor. As a result, the E1B55K-E4orf6-Cul2 complex from different types of human adenovirus showed different substrate specificity against p53 [128, 129], Mre11 [130], DNA ligase IV [131] and integrin α3 [126, 127, 132–135]. Among these substrates, DNA Ligase IV was the only universal substrate for all types of adenoviruses tested [126, 133]. In particular, Ad12 E4orf6 not only recruited the Cul2 ubiquitin ligase complex but also acted as a substrate receptor for the ATR activator protein topoisomerase-IIβ–binding protein 1 (TOPBP1). Ad12 E4orf6 could inhibit the ATR-dependent phosphorylation of CHK1 through promoting the proteasomal degradation of TOPBP1 in the absence of E1B55K [133, 136].

Epstein–Barr virus (EBV) is a human γ-herpesvirus, and it is able to induce several B cell and epithelial-cell malignancies. In viral life cycles, EBV periodically reactivates and replicates in a lytic manner [137]. Induction of the EBV lytic program was found to trigger a cellular DNA damage response via activating the ATM-dependent DNA damage signal transduction pathway [138]. This would induce apoptosis and limit viral replication by Chk2-mediated phosphorylation of p53 at its C-terminus [139, 140]. The EBV virus developed a method to circumvent this limitation. Bzlf1 protein of EBV had Cul2 and Cul5 boxes at its N-terminus and could form complexes with Cul2 and Cul5, and Bzlf1 recognized C-terminal phosphorylated p53 and induced p53 degradation to ensure efficient viral propagation [140, 141].

Human papillomaviruses (HPVs) are DNA viruses that specifically infect squamous epithelial cells Bernard HU2010. Among more than 120 different species identified so far, HPV16 was found in 50 % of cervical cancers [142]. E7 oncoprotein of HPV16 was necessary for the induction and maintenance of the oncogenic transformation [143]. HPV16 E7 was found to form a complex with Cul2 via an incomplete Cul2 box, and it bound and promoted the degradation of a hypophosphorylated form of the retinoblastoma tumor suppressor (RB1) [144–146]. This allowed RB1–E2F complexes to dissociate and the G1-S phase transition to proceed, allowing the replication of the viral DNA in differentiated host cells [147, 148]. In addition, Zyg-11 related cell cycle regulator (Zer1, also known as Zyg11bl) was required for the binding of HPV16 E7 to Cul2 and the destabilization of RB1 in HPV16 E7-expressing cells [149].

The viral infectivity factor (Vif) from human immunodeficiency virus type 1 (HIV-1) and simian immunodeficiency virus (SIV) could form a CRL5 E3 ubiquitin ligase complex to degrade host antiviral APOBEC3 (A3) proteins, so the HIV-1 could escape from A3-mediated host antiviral defense [150]. Similarly, Vif from bovine immunodeficiency virus (BIV) interacted with Cul2, Elongin B/C and Rbx1, instead of Cul5 and Rbx2 in HIV, to form a CRL2 E3 ubiquitin ligase. This complex was reported to degrade the bovine A3 proteins (A3Z2Z3 and A3Z3) [104, 151]. Consistently, BIV Vif with mutations in the BC box or putative VHL box, which failed interact with Elongin B/C or Cul2, respectively, lost the ability to regulate bovine A3 proteins [104].

## Conclusions

Among CRLs, Cul2 based E3 ligase complexes had a similar structure and binding partners with Cul5 based E3 ligase complexes, and both belonged to ECS family [3]. The substrate recognition receptor of Cul2 complex generally contained a VHL box, which contained a BC box and a Cullin box, and was very similar to SOCS box in Cul5 complex. Recent crystal structure analysis revealed the differences between CRL2 and CRL5 complexes, and indicated the possibility of fine-tuning CRL2 activity [17]. The activity of CRL2 can be regulated by neddylation on a key residue on Cul2 [24, 26]. Through various substrate receptors, CRL2 complexes recognize a number of substrates and regulate their protein stability and function through polyubiquitination (Table 1). Defects in various CRL2 complexes led to cancer and other human disease through abnormal stabilization and enhanced activity of their protein substrates. Inhibiting the activities of the substrates or those of their downstream effectors have shown clinical efficacy. As different viral proteins co-opt Cul2 to evade host defense, inhibiting their activities might help us fight various viral infections. Thus through better understanding of the biology of CRL2 complexes, we can devise and develop new therapeutic strategies against cancers, inherited diseases and viral infections caused by dysregulated CRL2 complexes.

## Abbreviations

CRLs: Cullin-RING E3 ubiquitin ligase complexes
Cul2: Cullin-2
Rbx1: RING protein
ECS: Elongin B and C-Cul2 or Cul5-SOCS box protein
VHL: von Hippel–Lindau
HIFα: α subunits of hypoxia inducible factor
CH: cullin homology
Cul5: Cullin-5
LRR-1: Leucine-Rich Repeat Protein -1
FEM-1: Feminization-1
SOCS: Suppressor of Cytokine Signaling
RCC: renal cell carcinoma
Csn5: COP9 signalosome complex subunit 5
Cand1: Cullin-Associated and Neddylation-Dissociated 1
Dcnl1: Defective in Cullin Neddylation 1
ARNT: Aryl hydrocarbon Receptor Nuclear Translocator
VEGFA: vascular endothelial growth factor A
SLC2A1: solute carrier family 2 member 1, also called GLUT1
PDGFB: platelet-derived growth factor-β
PHD: prolyl hydroxylase
LOH: loss of heterozygosity
Spry2: Sprouty2
PKC: protein kinase C
EGFR: epidermal growth factor receptor
HTP-3: HORMA-domain protein
SNP: single nucleotide polymorphism
Prame: preferentially expressed antigen in melanoma
RAR: retinoic acid receptor
Rack1: Receptor for Activated C Kinase 1
WD-repeat: tryptophan-aspartate repeat
BimEL: Bcl-2-interacting mediator of cell death extra long
AR: androgen receptor
Klhdc2: Kelch domain containing 2
Zswim2: Zinc finger, SWIM-type containing 2
Mex: MEKK1-related protein X
SWIM: SWI2/SNF2 and MuD
NMDA: N-methyl-D-aspartate
Ad: adenovirus
EBV: Epstein–Barr virus
HPV: human papillomavirus
BIV: bovine immunodeficiency virus
E4orf6: early region 4 from open reading frame 6
TOPBP1: topoisomerase-IIβ–binding protein 1
RB1: retinoblastoma tumor suppressor
Zer1: Zyg-11 related cell cycle regulator
Vif: viral infectivity factor
HIV-1: human immunodeficiency virus type 1
SIV: simian immunodeficiency virus
